# Complementary Feeding in Western Cape, South Africa: Identifying Suboptimal Practices and Potential Targets for Intervention

**DOI:** 10.7759/cureus.46512

**Published:** 2023-10-05

**Authors:** Joanah M Ikobah, Jan Taminiau, Etienne Nel, Mary Fewtrell

**Affiliations:** 1 Division of Paediatric Gastroenterology, Hepatology and Nutrition, Department of Paediatrics, University of Calabar/University of Calabar Teaching Hospital, Calabar, NGA; 2 Division of Paediatric Gastroenterology, Hepatology and Nutrition, Department of Paediatrics, Tygerberg Academic Hospital, Stellenbosch University, Cape Town, ZAF; 3 Department of Paediatric Gastroenterology, Academic Medical Center, Amsterdam, NLD; 4 Department of Population, Policy and Practice Research and Teaching, University College London (UCL) Great Ormond Street Institute of Child Health, London, GBR; 5 Department of Paediatrics, Great Ormond Street Hospital for Children NHS Foundation Trust, London, GBR

**Keywords:** south africa, western cape province, breastfeeding, infants, complementary feeding

## Abstract

Introduction

Appropriate complementary feeding is important for the normal growth and development of children. This study aimed to describe the complementary feeding practices and identify suboptimal practices that would be possible targets for intervention to improve practices among mothers of infants aged six weeks to one year in Western Cape Province, South Africa.

Methods

This hospital-based cross-sectional study was conducted at the emergency unit of the Department of Paediatrics and Child Health of Tygerberg Academic Hospital, Cape Town, South Africa, between May 2017 and June 2017 among 110 mothers and their infants. Infants with minor ailments were included in the study. Patients requiring hospital admissions or severely ill infants requiring oxygen or ventilation, premature babies, and children with congenital anomalies were excluded from the study. The relationship between sociodemographic variables and the time of commencement of complementary food was described.

Results

The mean age of infants was 6.4±3.2 months, while the mean age of mothers was 27.6±5.5 years. On average, the age at introduction of complementary food to infants was 2.17±1.50 months. Among the complementary foods given to infants less than six months of age, cereals were the most commonly introduced (76.5%), while the least were sweet beverages (5.9%). Maternal age ≤ 34 years and first-born infant were significantly associated with early commencement of complementary food before six months of age (p=0.042 and p=0.032, respectively).

Conclusion

This study indicated that commencement of complementary food as early as two months of age with the use of non-nutritious and inappropriate food remains a significant problem in the region. There is a need for further education of mothers on appropriate complementary feeding practices given the importance of complementary feeding practices to the optimum growth and development of children.

## Introduction

Appropriate complementary feeding is important to maintain health and normal development in children [1]. Complementary feeding period is defined as “the period during which other foods or liquids are provided along with breastmilk,” and complementary foods are “any nutrient-containing foods or liquids other than breast milk given to young children during the period of complementary feeding” [1]. Human milk alone does not meet the full nutritional requirements of infants after six months of age. Nutritious complementary feeds introduced into the diets of infants older than six months are important to achieve proper growth and development [1].

The incidence of malnutrition rises sharply from the age of 6-18 months in most developing countries, and inappropriate complementary feeding in infancy is a major contributor to malnutrition in young children [1]. Optimal complementary feeding together with breastfeeding may largely prevent severe malnutrition in developing countries [2]. About 40% of infants worldwide are exclusively breastfed for the first six months of life, and two-thirds are introduced to solid foods at the appropriate age [3]. However, only about 33% of children between the ages of six and 23 months receive optimal complementary feeding in terms of the quantity of food, frequency of feeding, and consistency of diet received [4].

The South African Demographic and Health Survey (SADHS) of 2003 showed that the exclusive breastfeeding (EBF) rate was 8.3% for infants less than six months and 0.4% for infants 6-9 months [5]. However, the 2016 SADHS, carried out 13 years later, showed that 32% of children under six months of age across South Africa were exclusively breastfed. This increase in the breastfeeding rate may reflect an increased awareness and acceptance of exclusive breastfeeding for children less than six months of life. About 25% of children were not breastfed at all [6]. Furthermore, it is concerning that 35%-50% of mothers in South Africa discontinue all breastfeeding within three months post-delivery, often introducing complementary foods as early as six weeks of age [7,8]. Additionally, these early complementary diets often lack proper nutrient density [7,8], and non-nutritious foods are still prevalent as complementary feeds in many regions of sub-Saharan Africa [7,9].

This study aimed to describe complementary feeding practices and identify risk factors for inappropriate practices in mothers presenting with their infants in the Western Cape Province of South Africa and to guide the identification of groups who should be targeted for advice.

## Materials and methods

Study setting

This study was conducted in the children emergency unit of the Department of Paediatrics and Child Health of Tygerberg Academic Hospital, Cape Town, South Africa, between May 2017 and June 2017. The emergency care unit at Tygerberg Hospital also provides a walk-in service for children who live in the close vicinity of the hospital. These children usually have minor complaints and are seen as outpatients. Participants were recruited from this patient group.

Study design

This was a cross-sectional study.

Study participants

One hundred ten (110) mothers of infants aged six weeks to one year who gave consent and met inclusion criteria, that is, children with minor illnesses, were sequentially enrolled in the study. Patients requiring hospital admissions or severely ill infants requiring oxygen or ventilation, premature babies, and children with congenital anomalies were excluded from the study.

Data collection

A semi-structured interviewer-administered questionnaire was administered to mothers. The primary language spoken by parents during the interview was English. The questionnaire was designed and adapted to the types of foods used locally. Common foods used locally include homemade cereals, maize-based porridge, green vegetables, yellow vegetables, yoghurt, chips, red meat, white meat, tea, and sweet beverages. A checklist of these foods was given to mothers to tick as appropriate. Information on socioeconomic and demographic characteristics of the family and feeding practice-related variables were assessed, including early initiation of breastfeeding (i.e., breastfeeding within one hour after birth), exclusive breastfeeding (EBF) under six months, time of introduction of solid, semisolid, or soft foods, and types of food given. The World Health Organization (WHO) definition of exclusive breastfeeding for six months was used in this study [10].

Ethical considerations

Ethical approval was obtained from the Health Research Ethics Committee of Stellenbosch University and Tygerberg Academic Hospital (N17/03/026). Participating mothers gave written informed consent.

Statistical analysis

Variables that followed a normal distribution were described with means and standard deviations in parenthesis, while variables that did not follow a normal distribution were described with medians and interquartile ranges in parenthesis. A test of association of categorical variables was done using the chi-square test. p-value < 0.05 was considered significant. A univariate analysis was used to explore associations between complementary feeding practice and potential indicators.

## Results

General characteristics of the study population

**Figure 1:**
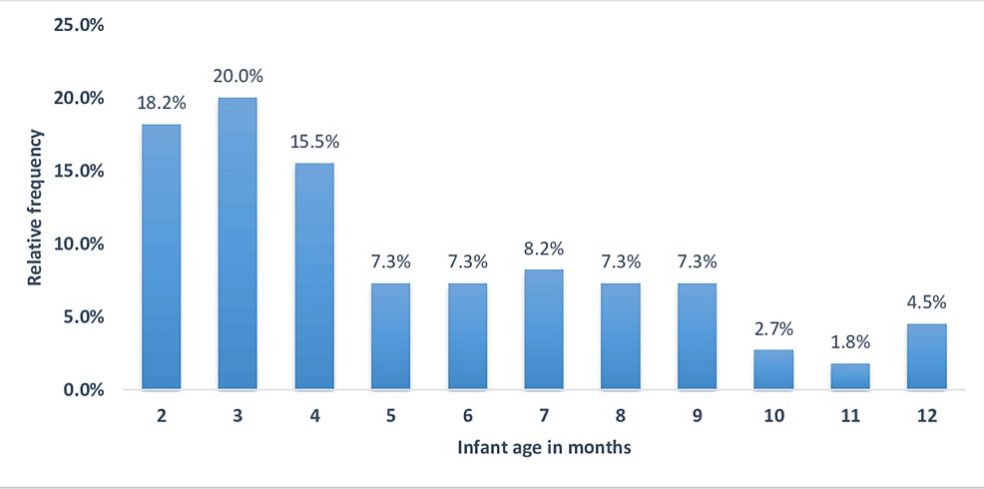
Frequency of infant age distribution

A total of 110 mothers and their infants were enrolled in the study, of which 64 (58.2%) of the infants were males. The mean age of infants was 6.4±3.2 months. Figure 1 shows the frequency distribution of infant ages.

The mean age of mothers was 27.6±5.5 years. Over half of the parents in the study lived together (57.3%); 40% were from a nuclear family setting, and 75.5% of the mothers did not work after delivery. Furthermore, 95.5% and 85.5% of mothers and fathers had secondary or higher educational levels, respectively. Most infants were of the second to fourth birth order (74 (67.3%)), with 32 (29.1%) firstborns (Table 1 and Table 2).

**Table 1:** Sociodemographic characteristics of mothers

Variable	Frequency	Percentage (%)
Age group (years)		
15-24	33	30
25-34	64	58.2
≥35	13	11.8
Mother’s education		
No formal/primary	4	3.6
Secondary and above	105	95.5
Unknown	1	0.9
Mother working after delivery		
Yes	27	24.5
No	83	75.5

**Table 2:** Sociodemographic and family characteristics of infants

Variable	Frequency	Percentage (%)
Sex of child		
Male	64	58.2
Female	46	41.8
Father’s education		
No formal/primary	2	1.8
Secondary and above	94	85.5
Unknown	14	12.7
Parents living together		
Yes	63	57.3
No	43	39.1
No response	4	3.6
Family setting		
Nuclear	44	40
Extended	19	17.3
No response	47	42.7
Birth order		
First	32	29.1
Second to fourth	74	67.3
Fifth and above	4	3.6

Breastfeeding practices

Timely initiation of breastfeeding within one hour of life occurred in 79 (72.4%) of the infants, and 83 (76.1%) were ever breastfed. Among those breastfed, the exclusive breastfeeding rate differed according to age in months ranging from 85% for infants at two months of age, 73% at four months, and 75% at five months. Twenty-seven (23.9%) infants were not breastfed at all.

Complementary feeding practices

Age at the Introduction of Complementary Feeds

**Figure 2:**
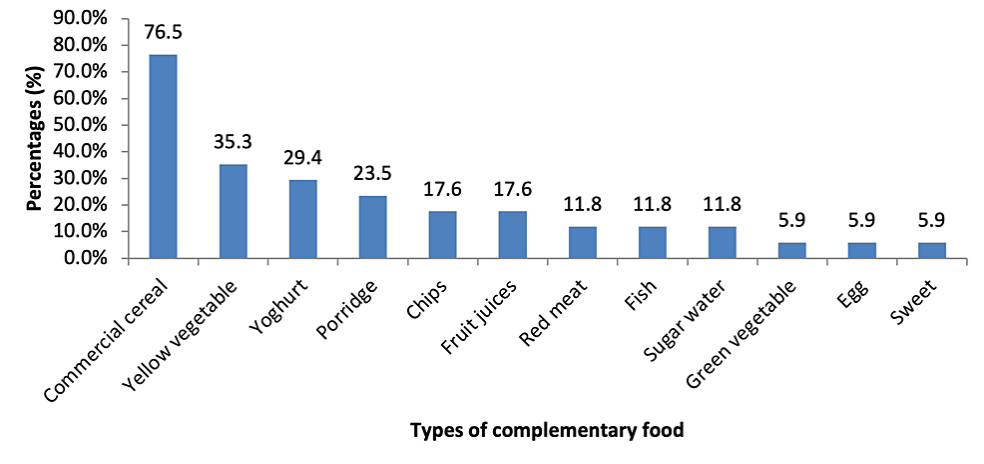
Complementary food given to infants aged less than six months

The mean age at the introduction of complementary food was 2.17±1.50 months. Early introduction of complementary food before six months of age was seen in 37 (34%) of the infants. Figure 2 shows the types of complementary food given to infants who were aged less than six months. The most common complementary food was commercial cereal given to 76.5% of infants, yellow vegetable (35.3%), yoghurt (29.4%), porridge (23.5%), chips (17.6%), fruit juices (17.6%), and sweet beverages (5.9%).

Relationship Between Sociodemographic Variables and Time of Commencement of Complementary Feeding

Younger mothers (34 years of age and less) and firstborn children were significantly associated with commencement of complementary food before six months of age (p=0.042 and p=0.032, respectively). Mother’s education, father’s education, number of siblings, family setting, mothers working after delivery, and parents living together were not significantly associated with complementary feeding practices (Table 3).

**Table 3:** Relationship between sociodemographic variables and time of commencement of complementary feed *Significant p-value **Fisher’s exact probability test

Variable	Time of commencement of complementary feed		X^2^ test	p-value
	<6 months (%)	≥6 months (%)		
Mother’s age group (years)				
15-24	22 (91.7)	2 (8.3)	6.34	0.042*
25-34	45 (93.8)	3 (6.2)		
≥35	7 (63.6)	4 (36.4)		
Mother’s education				
No formal/primary	2 (100)	0 (0)	0.25	1.000**
Secondary and above	71 (88.8)	9 (11.2)		
Father’s education				
No formal/primary	1 (50)	1 (50)	2.87	0.224**
Secondary and above	66 (89.2)	8 (10.8)		
Birth order				
First	22 (100)	0 (0)	6.91	0.032*
Second to fourth	49 (84.5)	9 (15.5)		
Fifth and above	3 (100)	0 (0)		
Number of siblings				
1	24 (100)	0 (0)	4.18	0.053**
2-3	49 (84.5)	9 (15.5)		
Family setting				
Nuclear	29 (80.6)	7 (19.4)	0.80	0.659**
Extended	11 (91.7)	1 (8.3)		
Mother working after delivery				
Yes	20 (95.2)	1 (4.8)	1.08	0.437**
No	54 (87.1)	8 (12.9)		
Parents living together				
Yes	40 (83.3)	8 (16.7)	3.37	0.081**
No	30 (96.8)	(3.2)		

Use of Non-prescribed Medications

The proportion of infants who received non-prescribed medications in the first six months of life was 51%. The most common medications given were gripe water and multivitamin syrups. No harmful medications were given.

## Discussion

Early initiation of breastfeeding within one hour of birth in this study was observed in 72.4% of infants. This rate is higher than the United Nations Children’s Fund (UNICEF) global database rate of 45% [11], the South African rate of 63% based on the 2015 UNICEF data [10], and the rate of 44.6% in Calabar, Nigeria [12]. This high rate shows that there is an increase in the awareness and importance of breastfeeding among mothers of infants studied. The ever-breastfed rate of 76.1% was lower than the 99% rate seen in rural Ga-Rankuwa, South Africa [13]. There was a decline in the breastfeeding rate as the age of the infants increased.

The introduction of complementary food occurred as early as two months of age in this study. Kruger et al. [8] and MacIntyre et al. [13] documented the commencement of complementary food as early as one month and six weeks of life, respectively, in South Africa. Ikobah et al. [12] documented an early commencement of complementary food at one month of life in Calabar, Nigeria. Studies in Tanzania [14], Kenya [15], and Malawi [16] have shown early commencement of complementary feed at ages 2-3 months of life. Early initiation of complementary feeding remains a common practice in developed and developing countries [17-19]. Although anecdotally noted in this study and not formally asked, most mothers perceive insufficient breast milk and babies not feeling satisfied as reasons for early commencement of complementary feeding. Although not measured in the mothers in this study, maternal nutritional status could theoretically influence the adequacy of breast milk or the success of breastfeeding. In the SADHS 2016 survey, only 3% of women aged 15 years and above were underweight in South Africa and 68% were overweight and obese. About 40% of young women aged 15-24 years of age were overweight or obese. The prevalence of anemia in women aged 15 years and above was 31%, and this was mostly mild anemia, most common in pregnant women. Food consumed during the day and night before the survey showed that 51% of women consumed fruits and 64% vegetables (excluding potatoes) [6].

Non-nutritious food and drinks were frequently offered to infants before the age of six months. These included juices, tea, sugar water, and chips. This has been documented by Faber et al. [9] in KwaZulu-Natal, South Africa, showing the poor dietary diversity and low nutrient density of complementary feeds served to children. This may explain the high burden of undernutrition in developing countries with increased morbidity, mortality, and long-term sequelae including short stature, poor school performance, and impaired intellectual and social development [20,21].

The study revealed that the mother’s age and birth order were factors significantly associated with early initiation of complementary feeding practice. Younger mothers in this study were more likely to introduce complementary feed before six months of age. The BeeBOFT study among Dutch infants showed that younger maternal age was independently associated with the early commencement of complementary food [19]. Mothers with firstborn children all practiced early commencement of complementary feeding before six months of age. This may be due to the inexperience of mothers influencing the practice of infant feeding. This finding is different from the study from Kemba Woreda in Southern Ethiopia, which showed mothers with children in the first birth order being significantly associated with appropriate initiation of complementary feeding [22].

In this study, there was no association of maternal education with complementary feeding practice. This may be due to the relatively small sample size in this study, and most parents were generally well-educated with secondary school or higher educational qualifications. This may have led to insufficient variation within the sample size to pick up an association. Mother’s occupation, father’s occupation, mother’s age, and parents living together were not significantly associated with complementary feeding practice in this study population.

In this study, 51% of the infants were given over-the-counter medications in the first six months of life. The commonest medication given was gripe water. It was believed the medication helps calm abdominal colic in infants. Some gripe waters available in this region do contain alcohol; however, the type of gripe water was not recorded in the study. Infants who participated in this study did not receive treatment from herbalists or traditional healers.

The limitation of this present study is the recall bias that may be present due to self-reporting of the timing of the introduction of complementary food. This, however, is reduced as the ages of children in the study are within the first year of life. Furthermore, this was a cross-sectional study, which limits the ability to draw conclusions about the age-related differences in feeding, as the same infants are not studied at different ages.

## Conclusions

Early introduction of complementary feeding, occurring before six months of age, was notably high within the study population. Additionally, the persistent use of non-nutritious and inappropriate foods remains a significant concern in this region. It is imperative for healthcare professionals to engage in ongoing education of mothers on appropriate infant nutrition, particularly targeting younger and first-time mothers. Such educational efforts are vital, as they can promote and instill appropriate complementary feeding practices. These practices are crucial for ensuring the optimal growth and development of children, underscoring the importance of continued support and guidance in this regard.
